# Current Affairs: A Mutation's-Eye View of Voltage Gating

**DOI:** 10.1371/journal.pbio.1000314

**Published:** 2010-02-23

**Authors:** Mary Hoff

**Affiliations:** Freelance Science Writer, Stillwater, Minnesota, United States of America

Like security guards screening guests at a celebrity event, gated ion channels embedded in the membranes of cells determine what molecules may pass through the membrane, and when they may do so. In the case of ligand-gated ion channels, the cue for opening the gate is the binding of a signaling molecule (ligand)—often a nucleotide or phospholipid—to an exposed site on the channel.

Another type of channel, known as voltage-gated, opens on a different cue. Rather than responding to the presence of a lock-and-key–like ligand, voltage-gated channels open when a part of the channel, known as the voltage-sensing domain (VSD), detects a change in potential across the membrane that causes a change from a conformation that prohibits passage of molecules to an open conformation that lets the molecules of choice move through.

Critical to multiple physiological functions and involved in genetic diseases such as epilepsies, ataxias, and cardiac arrhythmias, voltage gating is currently a subject of much study. And the more it is studied, the more complex it appears: Some channels with VSDs aren't gated by voltage, for instance, and some voltage-dependent channels don't have VSDs. Additional uncertainty arises from variations in coupling between the VSD and the channel pore. In many voltage-sensitive potassium K^+^ channels, the probability of the channel being open (Po) is strongly influenced by membrane potential, with Po being close to 0 at negative voltages and close to 1 when the membrane is depolarized. In others, however, the channel can have a measurable Po even at negative voltages.

In their efforts to unravel the mysteries and mechanisms behind channel gating, Harley Kurata, Colin Nichols, and colleagues discovered a surprising mutation of the ligand-gated K^+^ channel Kir6.2, in this issue of *PLoS Biology*. Referred to as Kir6.2[L157E], the mutant varies from the wild type by the substitution of a single amino acid at residue 157 on one of the polypeptide components. Remarkably, it gives the erstwhile ligand-gated channel the ability to respond to voltage cues as well, even though it lacks a VSD.

Recognizing in Kir6.2[L157E] a valuable tool for exploring the working of voltage gating, the researchers decided to analyze its behavior under various conditions. Confirming that voltage was really regulating the channel open probability, the researchers next manipulated voltage, ligand, and permeant ion concentrations to see the effect on gating in the mutant and wild-type channels. They discovered that the concentration of K^+^ inside the cell hugely influences this gating in Kir6.2[L157E]. Low K^+^ inside the cell increased the odds the channel would be open (as it does in wild type to a lesser extent), a condition that is reversed by introducing a positive charge at the mutation site (i.e., Kir6.2[L157K]). Intracellular K^+^ (and even Na^+^) affect channel interaction with the Kir6.2 ligand PIP_2_ as well, showing interesting interactions between the two types of gating exhibited by the mutant channel. In the presence of PIP_2_, a membrane lipid that promotes the opening of the channel, the voltage dependence disappeared. When PIP_2_ was reduced again, the voltage dependence reemerged.

After revealing an underlying voltage-dependent gating in a ligand-gated channel and demonstrating that voltage-dependent gating and ligand-dependent gating do not function independently, the researchers concluded that voltage gating and ligand gating may be more closely linked than was previously thought, with both operating on the same gate. They propose a simple but elegant model in which the four helices that line the channel and provide the gate are dragged closer or driven apart by the attractive or repulsive effects of ions located within the pore at the level of residue 157. Thus, in the absence of cations in the inner cavity of the channel, the two negative charges of the ions repel each other and the channel opens. When cations occupy the cavity, they attract the negative charges and the channel closes.

Overall, this study provides valuable new insights into voltage-dependent gating by demonstrating a mechanism by which intracellular K^+^ ions can influence gating, suggesting that voltage-dependent gating in conventional voltage-gated channels may be influenced not only by the voltage-sensing domain, but to other structural motifs common to voltage-gated channels and ligand-gated channels.

**Kurata HT, Rapedius M, Kleinman MJ, Baukrowitz TH, Nichols CG (2010) Voltage-Dependent Gating in a “Voltage Sensor-Less” Ion Channel. doi:10.1371/journal.pbio.1000315**

**Figure:**
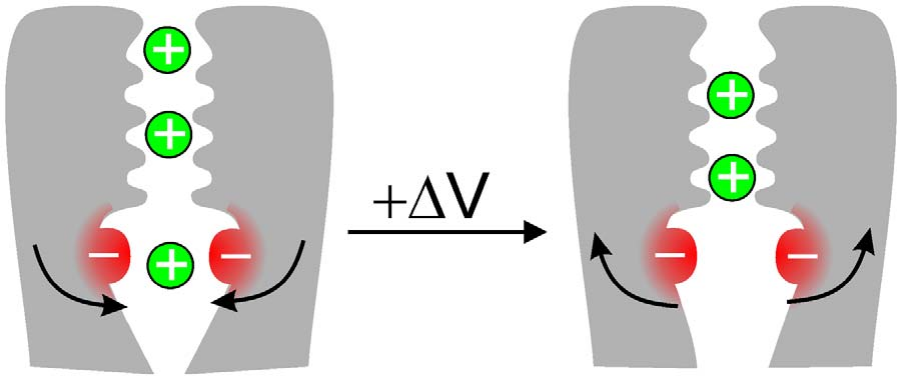
A novel mechanism of voltage gating has been identified in a ligand-gated channel whereby voltage-dependent occupancy of the inner cavity by permeant ions influences gating by electrostatic effects on pore lining helices.

